# Endometriosis and COVID-19: A Systematic Review and Meta-Analysis

**DOI:** 10.3390/ijms232112951

**Published:** 2022-10-26

**Authors:** Ziyaana Kabani, Maria E. Ramos-Nino, Prakash V. A. K. Ramdass

**Affiliations:** 1 Department of Public Health and Preventive Medicine, St. George’s University School of Medicine, St. George FZ818, Grenada; 2 Department of Microbiology, Immunology, and Pharmacology, St. George’s University School of Medicine, St. George FZ818, Grenada

**Keywords:** endometriosis, COVID-19, SARS-CoV-2, dysmenorrhea, pelvic pain, anxiety, depression

## Abstract

Endometriosis is defined as ectopic endometrial tissues dispersed outside the endometrium. This can cause disruption in hormonal and immunological processes, which may increase susceptibility to SARS-CoV-2 infection. Worsening of endometriosis symptoms may occur as a result of this infection. The aim of our review was to estimate the pooled prevalence of SARS-CoV-2 infection and the health impacts of the COVID-19 pandemic in endometriosis patients. We conducted a systematic review and meta-analysis. MEDLINE, Science Direct, Scopus, and Google Scholar databases were searched, using the keywords: (endometriosis) AND (COVID-19 OR SARS-CoV-2). Forest plots and pooled estimates were created using the Open Meta Analyst software. After screening 474 articles, 19 studies met the eligibility criteria for the systematic review, and 15 studies were included in the meta-analyses. A total of 17,799 patients were analyzed. The pooled prevalence of SARS-CoV-2 infection in endometriosis patients was 7.5%. Pooled estimates for the health impacts were 47.2% for decreased access to medical care, 49.3% increase in dysmenorrhea, 75% increase in anxiety, 59.4% increase in depression, and 68.9% increase in fatigue. Endometriosis patients were undeniably impacted by the COVID-19 pandemic, which caused the worsening of symptoms such as dysmenorrhea, pelvic pain, anxiety, depression, and fatigue.

## 1. Introduction

Endometriosis is one of the most frequent benign gynecological conditions diagnosed in premenopausal women, with an estimated world-wide prevalence ranging from 16–20% [1]. Millions of these patients are thought to have been impacted by the COVID-19 pandemic [2], and estimates suggest that 6.2% of them were infected with Severe Acute Respiratory Syndrome Corona Virus-2 (SARS-CoV-2) [3].

Histologically, endometriosis is defined as the presence of ectopic endometrial tissues dispersed in areas outside the endometrium [4], and is symptomatically characterized by pelvic pain, dysmenorrhea, dyspareunia, dyschezia, and infertility [5]. These symptoms are thought to occur as a result of disturbance in hormonal [6], neurological [7], and immunological functioning [8]. Disruption in these processes is believed to contribute to increase susceptibility of SARS-CoV-2 infection in endometriosis patients, which may in turn result in the worsening of symptoms. While the exact mechanism of symptom worsening is unclear, there is some evidence suggesting SARS-CoV-2 is involved [9].

Thus, the aim of this systematic review and meta-analysis was to estimate the pooled prevalence of SARS-CoV-2 in endometriosis patients, and to determine the risk of SARS-CoV-2 infection in endometriosis patients. In addition, we wanted to estimate the health impacts in endometriosis patients as a consequence of the COVID-19 pandemic.

## 2. Results

### 2.1. Characteristics of Identified Studies

**Figure 1:**
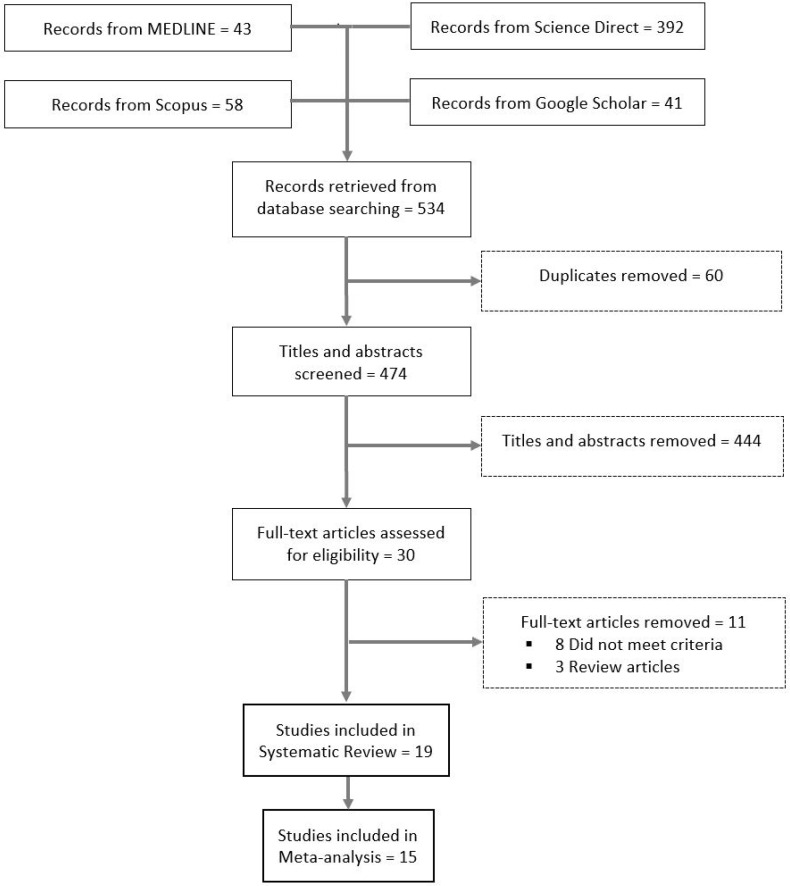
Flow diagram of the included studies.

As illustrated in Figure 1, the titles and abstracts of 474 articles were screened for eligibility, of which 30 met the eligibility criteria for full-text review. After further screening, 19 studies met the inclusion criteria for the systematic review.

Characteristics of the 19 included studies appear in Table 1. Quantitative data were available for only 15 studies, which were included in the meta-analyses. However, not all 15 studies had quantitative data for all the variables we assessed. Thus, meta-analyses of the included studies ranged from 2 (risk of COVID-19 in endometriosis patients) to 10 (decreased access to medical care for endometriosis patients). Individual patient data were obtained on 17,799 patients. The four studies not included in the meta-analysis were based on qualitative data [10,11,12,13]. In addition, four studies [3,14,15,16] appear in both the meta-analysis on the prevalence of SARS-CoV-2 in endometriosis patients as well as the meta-analyses on the health impacts of COVID-19 in endometriosis patients.

### 2.2. COVID-19 in Endometriosis Patients

**Figure 2:**
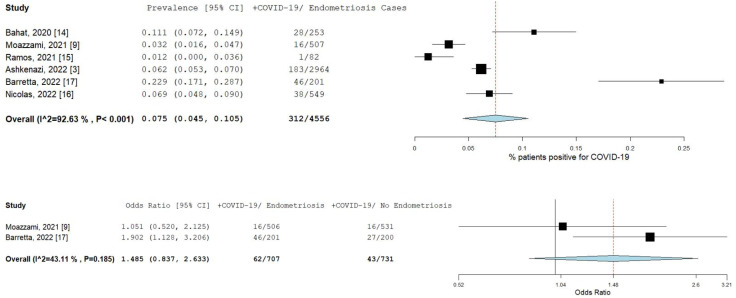
Prevalence and risk of COVID-19 in endometriosis patients. Square boxes represent individual studies; horizontal lines represent 95% confidence intervals (CIs); vertical dotted lines represent pooled estimate; and diamond-shaped figures represent 95% CIs of pooled estimate.

A total of six studies [3,9,14,15,16,17] comprising 4555 patients were included in the meta-analysis. Figure 2 illustrates the forest plot of the prevalence of COVID-19 in endometriosis patients, and the risk of COVID-19 infection in endometriosis patients. The pooled prevalence was 7.5% (95% CI [4.5–10.5]; I^2^ = 92.6%; *p* < 0.001). The prevalence of individual studies ranged from 1.2% [15] to 22.9% [17]. Only two studies [9,17] had available data to estimate the odds ratio. The pooled odds ratio for these two studies was 1.5 (95% CI [0.8–2.6]; I^2^ = 43.1%; *p* = 0.185), as shown in the forest plot in Figure 2.

### 2.3. Impact of COVID-19 in Endometriosis Patients

Seventeen studies (shown in Table 1) investigated one or more health impacts of the pandemic. However, quantitative data for any health impact were available for only 13 studies. A total of 17,092 patients were included in the meta-analyses. Table 2 illustrates the 10 factors we examined that impacted endometriosis patients because of the pandemic. Fourteen studies (10 with quantitative data) assessed the decreased access to medical care.

**Figure 3:**
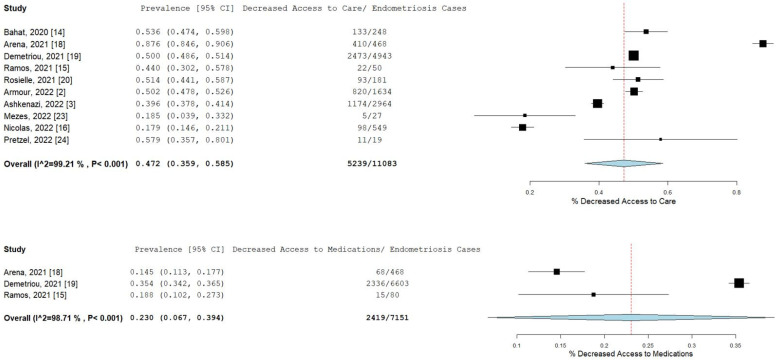
Pooled prevalence for decreased access to medical care and medications. Square boxes represent individual studies; horizontal lines represent 95% confidence intervals (CIs); vertical dotted lines represent pooled estimate; and diamond-shaped figures represent 95% CIs of pooled estimate.

Pooled prevalence rates are shown for decreased access to medical care and medications in Figure 3. Nearly half of the endometriosis patients stated that they had decreased access to their doctor or medical care during the pandemic (pooled prevalence = 47.2%; 95% CI [35.9–58.5]), while 23% (95% CI [10.2–27.3]) of patients related that they had decreased access to medications or treatment.

**Figure 4:**
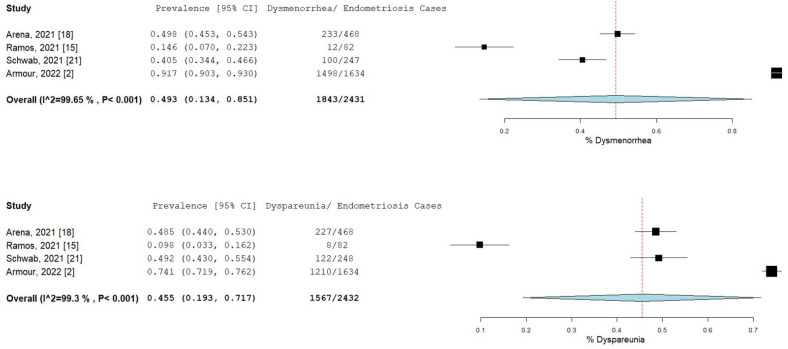
Pooled prevalence for increased dysmenorrhea and dyspareunia. Square boxes represent individual studies; horizontal lines represent 95% confidence intervals (CIs); vertical dotted lines represent pooled estimate; and diamond-shaped figures represent 95% CIs of pooled estimate.

**Figure 5:**
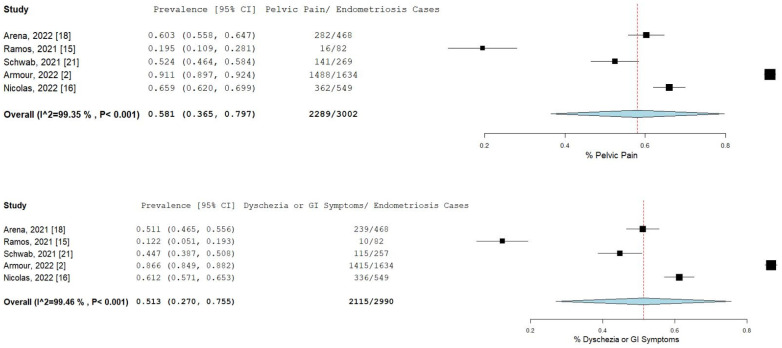
Pooled prevalence for increased pelvic pain and dyschezia and other GI symptoms. Square boxes represent individual studies; horizontal lines represent 95% confidence intervals (CIs); vertical dotted lines represent pooled estimate; and diamond-shaped figures represent 95% CIs of pooled estimate.

**Figure 6:**
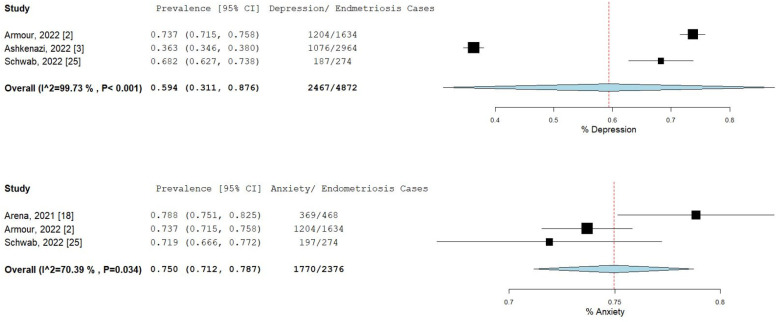
Pooled prevalence for increased depression and anxiety. Square boxes represent individual studies; horizontal lines represent 95% confidence intervals (CIs); vertical dotted lines represent pooled estimate; and diamond-shaped figures represent 95% CIs of pooled estimate.

**Figure 7:**
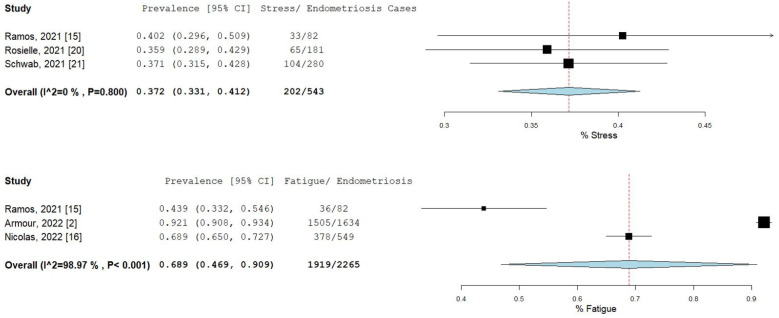
Pooled prevalence for increased stress and fatigue. Square boxes represent individual studies; horizontal lines represent 95% confidence intervals (CIs); vertical dotted lines represent pooled estimate; and diamond-shaped figures represent 95% CIs of pooled estimate.

The percentage and 95% CI of endometriosis patients who reported new, increased, or worsening symptoms were as follows: dysmenorrhea (49.3% [13.4–85.1]), dyspareunia (45.5% [19.3–71.7]) shown in Figure 4, pelvic pain (58.1% [36.5–79.7]), and dyschezia and other gastrointestinal symptoms (53.1% [27.0–75.5]) shown in Figure 5, depression (59.4% [31.1–87.6]) and anxiety (75.0% [71.2–78.7]) shown in Figure 6, and stress (37.2% [33.1–41.2]) and fatigue (68.9% [46.9–90.9]), shown in Figure 7.

### 2.4. Quality Assessment and Publication Bias

**Figure 8:**
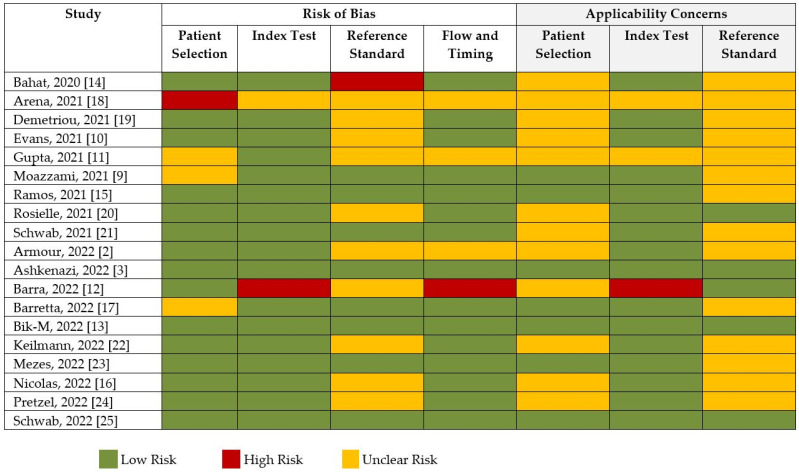
Risk of bias and quality assessment of studies according to QUADAS-2.

**Figure 9:**
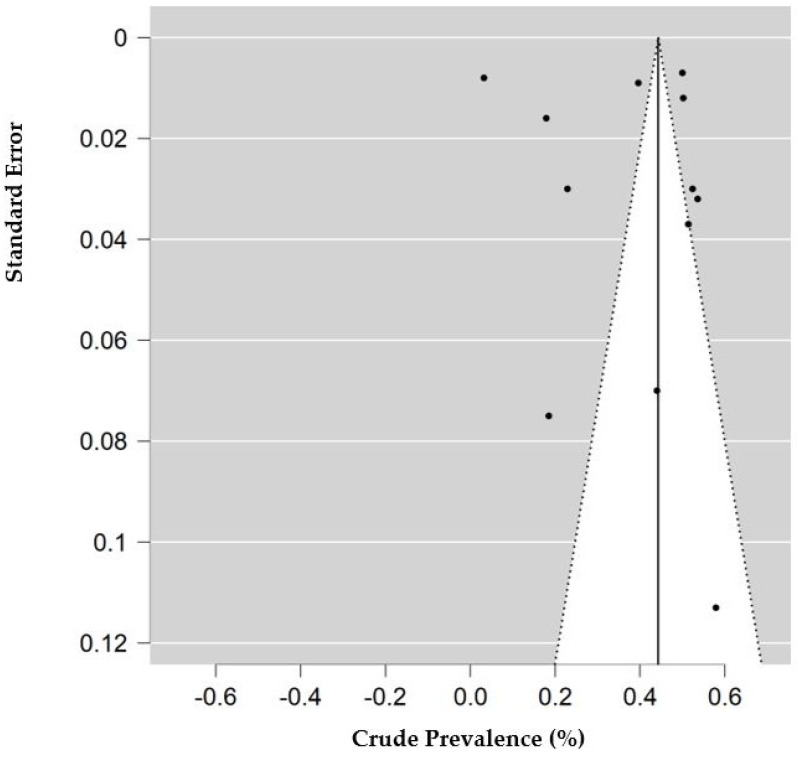
Funnel plot of studies with data for decreased access to medical care. Prevalence (percentage) of endometriosis patients who reported decreased access to medical care or medication. Standard error is calculated for the prevalence of each study.

The risk of bias and applicability assessment based on QUADRAS-2 for each study is shown in Figure 8. Many of the studies had a low risk of bias, followed by unclear risk of bias. Visual inspection of the funnel plot, shown in Figure 9, was inconclusive for risk of publication bias. However, objective analysis with Egger’s test (z = 0.131; *p* = 0.896) reveals there was no publication bias.

## 3. Discussion

The COVID-19 pandemic has affected millions of people around the world, with almost everyone being impacted negatively in some way [26]. Our meta-analysis shows that 7.5% of endometriosis patients were infected with SARS-CoV-2, and that endometriosis patients had an approximately 50% increased risk of acquiring COVID-19. When compared to the general population, the prevalence of COVID-19 in endometriosis patients seems to be higher. Moreover, endometriosis patients experienced increased pain symptoms such as dysmenorrhea [18], and increased mental health symptoms such as anxiety [2] and depression [3] as a consequence of the pandemic. Whether the worsening of these symptoms was directly as a result of SARS-CoV-2 infection, or indirectly because of decreased access to medical care and medication [19], or a combination of both, there is little evidence available to substantiate these observations.

Endometriosis is a disease due to endocrine and immune dysregulation [27], and its pathogenesis is poorly understood. However, Sampson first proposed the theory that endometriosis could be initiated due to retrograde menstruation and the dissemination of endometrial cells through the uterine tubes [28]. Nevertheless, this phenomenon occurs in most women of reproductive age, but the endometrial cells do not implant in the peritoneal cavity and are eliminated by the immune system by apoptosis [29]. In women with endometriosis, changes in cell-mediated and humoral immunity may contribute to the development of the disease [30].

Since an aberrant immune response in the peritoneal environment seems to be crucial for the proliferation of ectopic endometrial cells [29], the immune changes that follow SARS-CoV-2 infection could well contribute to endometriosis or vice versa. Immunologic and inflammatory changes that are observed in endometriosis include the following: decreased T-cell reactivity, NK cytotoxicity, increased antibody production, increased number and activation of peritoneal macrophages, and changes in inflammatory mediators [27,29,31]. An example of how COVID-19 and endometriosis could contribute to each other’s pathogenesis is via tumor necrosis factor α (TNF-α). Recent studies on COVID-19 suggest that cytokine release syndrome is associated with the severity of disease; this syndrome is characterized by increased TNF-α, interleukin (IL)-6, IL-2, IL-7, and IL-10 [32]. Cao and colleagues found that TNF-α plays a role in endometriosis [33], and its expression is increased in tissues of patients with COVID-19 [34].

To enter cells, SARS-CoV-2 uses its spike S protein to bind angiotensin-converting enzyme 2 (ACE2), which plays an important role in the renin-angiotensin-aldosterone system, and the transmembrane protease serine protease-2 (TMPRSS2) for S protein priming [35,36,37]. This process downregulates the expression of ACE2, leading to upregulation of the proinflammatory response induced by angiotensin II [38]. Other potential pathways of entry, such as the receptor Basigin (BSG/CD147) and proteases such as TMPRSS4, cathepsins B and L (CTSB and CTSL, respectively), FURIN, and MX dynamin-like GTPase 1 (MX1), are under investigation in relation to SARS-CoV-2 infectivity [39,40,41].

Henarejos-Castillo and collaborators analyzed the impact of SARS-CoV-2 infection on the gene expression for receptors in the endometrium and observed different expressions for the various receptors [42]. Additionally, gene expression for some of the receptors was found to increase with age, and expression varied throughout the different phases of the menstrual cycle [42]. Thus, the endometrium has an overall low risk of SARS-CoV-2 infection, due to the low expression of ACE2 [43] and intermediate expression of TMPRSS2 [42]. However, the risk changes with varying expression of these host receptors at specific stages of the menstrual cycle [42,44]. Nonetheless, their study suggests that low expression of ACE2 and TMPRSS2 in endometrial cells does not imply that other mechanisms are not involved in infectivity [44].

Remarkably, even if infection of endometrial cells by SARS-CoV-2 is unlikely, gene expression of their receptors is altered. A study by Miguel-Gomez et al. involving a cohort of 24 women with COVID-19 (*n* = 14) and without COVID-19 (*n* = 10), showed that even though SARS-CoV-2 was absent from the endometrial tissue in COVID-19 patients, there was alteration in gene expression for receptors in the endometrial tissue despite the absence of SARS-CoV-2 RNA [45]. In addition, from a clinical perspective, it remains unclear whether patients with thoracic endometriosis may have a higher risk of pulmonary disease or SARS-CoV-2 infection [46]. However, evidence from recent studies has shown an increased risk of SARS-CoV-2 infection in other common gynecological conditions such as endometrial hyperplasia and cancer [47], polycystic ovary syndrome [48], and breast cancer [49].

The explanation for the increase in clinical manifestations of endometriosis such as pelvic pain, dysmenorrhea, and dyspareunia during the COVID-19 pandemic is unclear. However, evidence related to these altered pain symptoms has pointed to the cascading of events in the renin-angiotensin–aldosterone system (RAAS), which leads to enhanced oxidative stress [50]. The binding of SARS-CoV-2 to ACE2 receptors causes the accumulation of angiotensin II, resulting in the impairment of the RAAS, which in turn generates enhanced oxidative stress, thereby producing inflammation, vasoconstriction, and endothelial dysfunction [51,52]. These resultant changes manifest as amplified nociceptive inflammatory pain [7]. Additionally, endometriosis patients are found to have elevated levels of proinflammatory factors such as IL-6, IL-8, TNF-α, and prostaglandin E2 [53]. Besides this, it has been well established that SARS-CoV-2 patients are observed to have high levels of IL-6, IL-8, and TNF-α [54]. Thus, SARS-CoV-2 infection intensifies an already proinflammatory state seen in endometriosis patients.

However, many of the endometriosis patients included in this analysis reported the worsening of existing symptoms [3,16] or the development of new symptoms [15], despite testing negative for SARS-CoV-2. Therefore, it can be postulated that the worsening of symptoms was also due to impacts of the COVID-19 pandemic, rather than solely the result of SARS-CoV-2 infection. Besides this, patients who experienced aggravated symptoms also reported decreased access to medical care and medications [3]. This was directly as a result of the global lockdown, which caused the unavailability of transportation, etc. [30]. Furthermore, the increased anxiety, depression, and stress reported in these patients could be explained by several mechanisms and factors. The chronic pain experienced by these patients causes alteration in neurotransmitters responsible for changes in mood [55], the chronic proinflammatory state causes an impairment of the blood–brain barrier, resulting in behavioral disturbance [56], and loneliness and isolation produces hormonal imbalance [56].

A study by Arena et al. Indicated that patients who reported an increase in anxiety were also shown to have increased stress, which was as a result of difficulty in obtaining hormonal therapy [22]. In addition, endometriosis patients were more likely to experience stress due to the cancellation or postponement of fertility treatment, or medical or surgical appointment [3]. Our results showed that the pooled estimate for anxiety in endometriosis patients was 75%, which was the highest proportion recorded when compared to all the other symptoms. Moreover, Barra and colleagues demonstrated that the majority of endometriosis patients with depression had moderate intensity, and that their Patient Health Questionnaire-9 (PHQ-9) and General Anxiety Disorder-7 (GAD-7) scores were significantly correlated [16]. Finally, univariate analysis revealed that endometriosis patients were twice as likely to experience fatigue when compared to healthy controls [20]. Thus, an increase in pain and worsening of mental health symptoms in endometriosis patients may be due to multiple factors as a result of SARS-CoV-2 infections and the effects of the COVID-19 pandemic.

Although most studies reported the negative impacts of the pandemic (such as increased symptoms or decreased access to medical care and medications), a study by Evans et al. reported some positive effects: approximately 12% of women with endometriosis related the benefits of working from home and the convenience of telehealth, which allowed for better symptom management [10]. However, these positive effects are not tabulated in our results. While our study reported the adverse impacts of the pandemic on endometriosis patients only, evidence has shown the far-reaching effects of the global pandemic on patients with other conditions as well [30].

## 4. Materials and Methods

This systematic review and meta-analysis was completed according to the PRISMA guidelines [57], and the protocol was pre-registered and published in PROSPERO (CRD42022356074) [58].

### 4.1. Data Sources and Search Strategy

We conducted a comprehensive search on MEDLINE, Science Direct, Scopus, and Google Scholar, using the following keywords: (endometriosis) AND (COVID-19 OR SARS-CoV-2). The search included studies published from 1 January 2020 through 26 August 2022. The language was restricted to English.

### 4.2. Study Selection and Eligibility Criteria

Citation files for the databases searched were imported into Zotero and duplicates were removed. The titles and abstracts were screened by two reviewers (Z.K. and P.R.) for eligibility, after which the full text of articles meeting the inclusion criteria was further examined for inclusion in this review. Any differences in study eligibility were resolved through discussion by the reviewers. Inclusion criteria were original, peer-reviewed studies (cohort studies, case-control studies, cross-sectional studies, mixed-methods, and randomized studies) that investigated the prevalence of SARS-CoV-2 in patients with endometriosis, and studies that examined the health impacts of the COVID-19 pandemic on endometriosis patients. We excluded case reports, cases series, review articles, abstracts, conference proceedings, and studies in which full-text articles were unavailable.

### 4.3. Study Outcomes, Data Extraction, and Quality Assessment

We examined two aspects of COVID-19 on endometriosis patients, whose diagnosis had to be confirmed surgically or clinically. A diagnosis of COVID-19 had to be confirmed by RT-PCR or antigen testing. Firstly, we analyzed the proportion of endometriosis patients who were tested positive for SARS-CoV-2. Secondly, we analyzed the health impacts that the COVID-19 pandemic had on endometriosis patients. The health impacts that we examined include access to medical care, access to medication, chronic pelvic pain, dysmenorrhea, dyspareunia, gastrointestinal symptoms such as dyschezia, fatigue, stress, anxiety, and depression.

The data for study characteristics, number of endometriosis patients, proportion of endometriosis patients according to SARS-CoV-2 status, and health impacts of the COVID-19 pandemics and their assessment tools, were extracted onto an Excel(R) spreadsheet. A quality assessment of the included studies was conducted using the QUADAS-2 [59] risk-of-bias assessment tool by two reviewers (Z.K. and P.R.).

### 4.4. Statistical Analysis

Pooled estimates and 95% confidence intervals (CI) were calculated for the prevalence of COVID-19 in endometriosis patients, along with the health impacts of the COVID-19 pandemic in endometriosis patients, using the generic inverse-variance method. We used the random effects method. The total prevalence was reported as a percentage among the included studies. The heterogeneity between studies was assessed using the I^2^ statistic. Studies with an I^2^ statistic > 50% were considered to have significant heterogeneity. Pooled analyses were considered statistically significant when the *p* value < 0.05. Open Meta Analyst software was used to create forest plots and analyze the included studies. Publication bias was examined by visually inspecting a funnel plot created by the JASP software, and by performing Egger’s test [60]. The forest plot was created only from studies with available quantitative data on access to medical care (10 studies) and medications (2 studies).

## 5. Conclusions

Our analysis suggests that endometriosis patients may have increased susceptibility to SARS-CoV-2 infection. However, due to insufficient data on homogenous groups, our study did not reach statistical significance for the risk estimate. Nevertheless, the prevalence of SARS-CoV-2 infection in endometriosis patients was substantial. Undeniably, endometriosis patients were negatively impacted regarding access to medical care during the COVID-19 pandemic. Moreover, a majority of patients experienced the worsening of pelvic pain, anxiety, depression, and fatigue, whereas approximately half of the patients reported increased dysmenorrhea, dyspareunia, and dyschezia.

## Figures and Tables

**Table 1:** Characteristics of the studies on the prevalence and impact of COVID-19 in endometriosis patients.

Prevalence of COVID-19					
**Study**	**Study Site**	Study Design	Sample Size	Endometriosis Cases (n)	Endometriosis Cases + for COVID-19 (n, %)
Bahat, 2020 [14]	Turkey	Cross-sectional	253	253	28 (11.1)
Moazzami, 2021 [9]	Iran	Case-control	1027	506	16 (3.2)
Ramos, 2021 [15]	Puerto Rico	Cross-sectional	82	82	1 (0.01)
Ashkenazi, 2022 [3]	International *	Cross-sectional	2964	2964	183 (6.2)
Barretta, 2022 [17]	Italy	Case-control	401	201	46 (22.9)
Nicolas, 2022 [16]	Spain	Prospective cohort	945	549	38 (6.9)
Impact of COVID-19					
**Study**	**Study Site**	**Study Design**	**Sample Size**	Assessment Tool	
Bahat, 2020 [14]	Turkey	Cross-sectional	253	EHP-5, GAD-7, STAI-Y6, IES-R	
Arena, 2021 [18]	Italy	Cross-sectional	468	-	
Demetriou, 2021 [19]	International ^★^	Cross-sectional	6729	Numerical rating scale	
Evans, 2021 [10]	Australia	Mixed-methods	162	-	
Gupta, 2021 [11]	USA	Retrospective cohort	221	Peritraumatic Distress Inventory	
Ramos, 2021 [15]	Puerto Rico	Cross-sectional	82	Fertility QOL ENDOCARE	
Rosielle, 2021 [20]	Netherlands	Cross-sectional	181	Visual Analog Pain Scale, PDI	
Schwab, 2021 [21]	Germany	Cross-sectional	285	Endometriosis Health Profile-30	
Armour, 2022 [2]	International †	Cross-sectional	1634	Endometriosis Health Profile-30	
Ashkenazi, 2022 [3]	International *	Cross-sectional	2964	GAD-7, PHQ-9	
Barra, 2022 [12]	Italy	Cross-sectional	-	-	
Keilmann, 2022 [22]	Germany	Retrospective cohort	3576	Depression, Anxiety and Stress Scale-21	
Bik-M, 2022 [13]	Australia	Prospective cohort	138	EQ-5D-3L	
Mezes, 2022 [23]	USA	Prospective cohort	27	Modified Symptom and QOL	
Nicolas, 2022 [16]	Spain	Prospective cohort	549	-	
Pretzel, 2022 [24]	USA	Cross-sectional	70	Visual Analog Pain Scale, PHQ-4	
Schwab, 2022 [25]	Germany	Cross-sectional	274	EHP-5, GAD-7, STAI-Y6, IES-R	

*—included 59 countries; ^★^—included 84 countries; †—included 46 countries. EHP-5—Endometriosis Health Profile-5; GAD-7—General Anxiety Disorder-7; STAI-Y6—Spielberger State-Trait Anxiety Inventory-Y6; QOL—Quality of Life; PHQ—Patient Health Questionnaire; EQ-5D-3L—European Quality of Life 5 Dimensions 3 Level Version. - Assessment tool not used or stated.

**Table 2:** Impacts of the COVID-19 pandemic on endometriosis patients.

Study	Access to Doctor/Care ↓	Access to Medication ↓	Chronic Pelvic Pain ↑	Dysmenorrhea ↑	Dyspareunia ↑	Dyschezia and Other GI Symptoms ↑	Fatigue ↑	Stress ↑	Anxiety/Worry ↑	Depression ↑
Bahat, 2020 [14]	✓	-	-	-	-	-	-	-	-	-
Arena, 2021 [18]	✓	✓	✓	✓	✓	✓	-	-	✓	-
Demetriou, 2021 [19]	✓	✓	-	-	-	-	-	-	-	-
Evans, 2021 [10]	✓	✓	-	✓	-	-	-	✓	✓	-
Gupta, 2021 [11]	✓	-	-	-	-	-	-	-	-	-
Ramos, 2021 [15]	✓	✓	✓	✓	✓	✓	✓	✓	✓	✓
Rosielle, 2021 [20]	✓	-	-	-	-	-	-	✓	-	-
Schwab, 2021 [21]	-	-	✓	✓	✓	✓	-	✓	-	-
Armour, 2022 [2]	✓	✓	✓	✓	✓	✓	✓	✓	✓	✓
Ashkenazi, 2022 [3]	✓	-	-	-	-	-	-	-	-	✓
Barra, 2022 [12]	✓	✓	✓	✓	✓	-	-	✓	✓	✓
Bik-M, 2022 [13]	-	-	-	-	-	-	-	✓	✓	✓
Keilmann, 2022 [22]	✓	-	-	-	-	-	-	-	-	-
Mezes, 2022 [23]	✓	-	-	-	-	-	-	-	-	-
Nicholas, 2022 [16]	✓	-	✓	-	-	✓	✓	-	-	-
Pretzel, 2022 [24]	✓	-	-	-	-	-	-	-	-	-
Schwab, 2022 [25]	-	-	-	-	-	-	-	-	✓	✓

↑ increased; ↓ decreased; ✓ assessed in the study; - not assessed in the study.

## Data Availability

Data can be found in the following databases: MEDLINE, Science direct, Scopus, and Google scholar.

## References

[1] Moradi Y., Shams-Beyranvand M., Khateri S., Gharahjeh S., Tehrani S., Varse F., Tiyuri A., Najmi Z. (2021). A Systematic Review on the Prevalence of Endometriosis in Women. Indian J. Med. Res..

[2] Armour M., Sinclair J., Cheng J., Davis P., Hameed A., Meegahapola H., Rajashekar K., Suresh S., Proudfoot A., Leonardi M. (2022). Endometriosis and Cannabis Consumption During the COVID-19 Pandemic: An International Cross-Sectional Survey. Cannabis Cannabinoid Res..

[3] Ashkenazi M.S., Huseby O.L., Kroken G., Soto-Mota A., Pents M., Loschiavo A., Lewandowska R., Tran G., Kwiatkowski S. (2022). COVID-19 Pandemic and the Consequential Effect on Patients with Endometriosis. Hum. Reprod. Open.

[4] Camboni A., Marbaix E. (2021). Ectopic Endometrium: The Pathologist’s Perspective. Int. J. Mol. Sci..

[5] Saunders P.T.K., Horne A.W. (2021). Endometriosis: Etiology, Pathobiology, and Therapeutic Prospects. Cell.

[6] Yilmaz B.D., Bulun S.E. (2019). Endometriosis and Nuclear Receptors. Hum. Reprod. Update.

[7] Gruber T.M., Mechsner S. (2021). Pathogenesis of Endometriosis: The Origin of Pain and Subfertility. Cells.

[8] Agostinis C., Balduit A., Mangogna A., Zito G., Romano F., Ricci G., Kishore U., Bulla R., Okró M. (2021). Immunological Basis of the Endometriosis: The Complement System as a Potential Therapeutic Target. Front. Immunol..

[9] Moazzami B., Chaichian S., Samie S., Zolbin M.M., Jesmi F., Akhlaghdoust M., Pishkuhi M.A., Mirshafiei Z.S., Khalilzadeh F., Safari D. (2021). Does Endometriosis Increase Susceptibility to COVID-19 Infections? A Case-Control Study in Women of Reproductive Age. BMC Women’s Health.

[10] Evans S., Dowding C., Druitt M., Mikocka-Walus A. (2021). “I’m in Iso All the Time Anyway”: A Mixed Methods Study on the Impact of COVID-19 on Women with Endometriosis. J. Psychosom. Res..

[11] Gupta S., Maghsoudlou P., Ajao M., Einarsson J.I., King L.P. (2021). Analysis of COVID-19 Response and Impact on Gynecologic Surgery at a Large Academic Hospital System. JSLS.

[12] Barra F., Lucia V., Rosa L., Vitale S.G., Commodari E., Altieri M., Scala C., Ferrero S. (2022). Psychological Status of Infertile Patients Who Had in Vitro Fertilization Treatment Interrupted or Postponed Due to COVID-19 Pandemic: A Cross-Sectional Study. J. Psychosom. Obstet. Gynecol..

[13] Bik-Multanowska K., Mikocka-Walus A., Fernando J., Westrupp E. (2022). Mental Distress of Parents with Chronic Diseases during the COVID-19 Pandemic in Australia: A Prospective Cohort Study. J. Psychosom. Res..

[14] Bahat P.Y., Kaya C., Selçuki N.F., Polat İ., Usta T., Oral E., Mah A. (2020). The COVID-19 Pandemic and Patients with Endometriosis: A Survey-Based Study Conducted in Turkey. Int. J. Gynecol. Obstet..

[15] Ramos-Echevarría P.M., Soto-Soto D.M., Torres-Reverón A., Appleyard C.B., Akkawi T., Barros-Cartagena B.D., López-Rodríguez V., Castro-Figueroa E.M., Flores-Caldera I. (2021). Impact of the Early COVID-19 Era on Endometriosis Patients: Symptoms, Stress, and Access to Care. J. Endometr. Pelvic Pain Disord..

[16] Nicolás I., Martínez-Zamora M.Á., Gracia M., Feixas G., Rius M., Carmona F. (2022). Impact of SARS-COV2 Pandemic on Patients with Endometriosis and Their Health Care. J. Women’s Health.

[17] Barretta M., Savasta F., Pietropaolo G., Barbasetti A., Barbera V., Vignali M. (2022). COVID-19 Susceptibility in Endometriosis Patients: A Case Control Study. Am. J. Reprod. Immunol..

[18] Arena A., Orsini B., Esposti E.D., Raimondo D., Lenzi J., Verrelli L., Iodice R., Casadio P., Seracchioli R. (2021). Effects of the SARS-CoV-2 Pandemic on Women Affected by Endometriosis: A Large Cross-Sectional Online Survey. Ann. Med..

[19] Demetriou L., Cox E., Lunde C.E., Becker C.M., Invitti A.L., Martínez-Burgo B., Kvaskoff M., Garbutt K., Evans E., Fox E. (2021). The Global Impact of COVID-19 on the Care of People With Endometriosis. Front. Glob. Women’s Health.

[20] Rosielle K., Bergwerff J., Schreurs A.M.F., Knijnenburg J., Bie B.D., Maas J.W.M., Nap A.W., van Wely M., Lambalk C.B., Goddijn M. (2021). The Impact of the COVID-19 Pandemic on Infertility Patients and Endometriosis Patients in the Netherlands. Reprod. Biomed. Online.

[21] Schwab R., Anić K., Stewen K., Schmidt M.W., Kalb S.R., Kottmann T., Brenner W., Domidian J.-S., Krajnak S., Battista M.J. (2021). Pain Experience and Social Support of Endometriosis Patients during the COVID-19 Pandemic in Germany—Results of a Web-Based Cross-Sectional Survey. PLoS ONE.

[22] Keilmann L., Beyer S., Meister S., Jegen M., Buschmann C., Schröder L., Keckstein S., Jeschke U., Burges A., Mahner S. (2022). Trends among Patients with Endometriosis over a 7-Year Period and the Impact of the COVID-19 Pandemic: Experience from an Academic High-Level Endometriosis Centre in Germany. Arch. Gynecol. Obstet..

[23] Mezes C., Klebanoff J.S., Grebenyuk E.W., Gobern J., Meske S.W., Amdur R., Moawad G.N. (2022). Virtual Postoperative Visits Following Robotic Gynecologic Surgery: A Study of Patient Satisfaction, Safety, and Feasibility. J. Robot. Surg..

[24] Pretzel S., Kuhn K., Pal L., Polotsky A., Taylor H.S., Zhang H., Robins J., Young S.L., Santoro N. (2022). Willingness of Women with Endometriosis Planning to Undergo IVF to Participate in a Randomized Clinical Trial and the Effects of the COVID-19 Pandemic on Potential Participation. Reprod. Sci..

[25] Schwab R., Stewen K., Ost L., Kottmann T., Theis S., Elger T., Schmidt M.W., Anic K., Kalb S.R., Brenner W. (2022). Predictors of Psychological Distress in Women with Endometriosis during the COVID-19 Pandemic. Int. J. Environ. Res. Public Health.

[26] Onyeaka H., Anumudu C.K., Al-Sharify Z.T., Egele-Godswill E., Mbaegbu P. (2021). COVID-19 Pandemic: A Review of the Global Lockdown and Its Far-Reaching Effects. Sci. Prog..

[27] Laganà A.S., Garzon S., Götte M., Viganò P., Franchi M., Ghezzi F., Martin D.C. (2019). The Pathogenesis of Endometriosis: Molecular and Cell Biology Insights. Int. J. Mol. Sci..

[28] Persoons E., Clercq K.D., den Eynde C.V., Pinto S.J.P.C., Luyten K., Bree R.V., Tomassetti C., Voets T., Vriens J. (2020). Mimicking Sampson’s Retrograde Menstrual Theory in Rats: A New Rat Model for Ongoing Endometriosis-Associated Pain. Int. J. Mol. Sci..

[29] Da Gama Coelho Riccio L., Santulli P., Marcellin L., Abrão M.S., Batteux F., Chapron C. (2018). Immunology of Endometriosis. Best Pract. Res. Clin. Obstet. Gynaecol..

[30] Björk E., Vinnars M.-T., Nagaev I., Nagaeva O., Lundin E., Ottander U., Mincheva-Nilsson L. (2020). Enhanced Local and Systemic Inflammatory Cytokine MRNA Expression in Women with Endometriosis Evokes Compensatory Adaptive Regulatory MRNA Response That Mediates Immune Suppression and Impairs Cytotoxicity. Am. J. Reprod. Immunol..

[31] Jiang L., Yan Y., Liu Z., Wang Y. (2016). Inflammation and Endometriosis. Front. Biosci..

[32] Guo Y., Hu K., Li Y., Lu C., Ling K., Cai C., Wang W., Ye D. (2022). Targeting TNF-α for COVID-19: Recent Advanced and Controversies. Front. Public Health.

[33] Cao X.L., Chai J., Yu Y.Y., Tian X., Zhao J.Y., Yu L.Y., Sun Z.G. (2020). Association of TNF-α Gene T-1031C Polymorphism with Endometriosis: A Meta-Analysis. Am. J. Reprod. Immunol..

[34] Popescu I., Snyder M.E., Iasella C.J., Hannan S.J., Koshy R., Burke R., Das A., Brown M.J., Lyons E.J., Lieber S.C. (2022). CD4+ T Cell Dysfunction in Severe COVID-19 Disease Is TNFα/TNFRI-Dependent. Am. J. Respir. Crit. Care Med..

[35] Hoffmann M., Kleine-Weber H., Schroeder S., Krüger N., Herrler T., Erichsen S., Schiergens T.S., Herrler G., Wu N.H., Nitsche A. (2020). SARS-CoV-2 Cell Entry Depends on ACE2 and TMPRSS2 and Is Blocked by a Clinically Proven Protease Inhibitor. Cell.

[36] Zhou P., Yang X.-L., Wang X.-G., Hu B., Zhang L., Zhang W., Si H.-R., Zhu Y., Li B., Huang C.-L. (2020). A Pneumonia Outbreak Associated with a New Coronavirus of Probable Bat Origin. Nature.

[37] Yan R., Zhang Y., Li Y., Xia L., Guo Y., Zhou Q. (2020). Structural Basis for the Recognition of SARS-CoV-2 by Full-Length Human ACE2. Science.

[38] Kai H., Kai M. (2020). Interactions of Coronaviruses with ACE2, Angiotensin II, and RAS Inhibitors-Lessons from Available Evidence and Insights into COVID-19. Hypertens. Res..

[39] Wang K., Chen W., Zhang Z., Deng Y., Lian J.-Q., Du P., Wei D., Zhang Y., Sun X.-X., Gong L. (2020). CD147-Spike Protein Is a Novel Route for SARS-CoV-2 Infection to Host Cells. Signal Transduct. Target. Ther..

[40] Zang R., Castro M.F.G., Mccune B.T., Zeng Q., Rothlauf P.W., Sonnek N.M., Liu Z., Brulois K.F., Wang X., Greenberg H.B. (2020). TMPRSS2 and TMPRSS4 Promote SARS-CoV-2 Infection of Human Small Intestinal Enterocytes. Sci. Immunol..

[41] Huang S., Fishell G. (2022). In SARS-CoV-2, Astrocytes Are in It for the Long Haul. Proc. Natl. Acad. Sci. USA.

[42] Henarejos-Castillo I., Sebastian-Leon P., Devesa-Peiro A., Pellicer A., Diaz-Gimeno P. (2020). SARS-CoV-2 Infection Risk Assessment in the Endometrium: Viral Infection-Related Gene Expression across the Menstrual Cycle. Fertil. Steril..

[43] Hikmet F., Méar L., Edvinsson Å., Micke P., Uhlén M., Lindsko C. (2020). The Protein Expression Profile of ACE2 in Human Tissues. Mol. Syst. Biol..

[44] Chadchan S.B., Popli P., Maurya V.K., Kommagani R. (2021). The SARS-CoV-2 Receptor, Angiotensin-Converting Enzyme 2, Is Required for Human Endometrial Stromal Cell Decidualization. Biol. Reprod..

[45] de Miguel-Gómez L., Romeu M., Castells-Ballester J., Pellicer N., Faus A., Mullor J.L., Pellicer A., Cervelló I. (2022). Undetectable Viral RNA from SARS-CoV-2 in Endometrial Biopsies from Women with COVID-19: A Preliminary Study. Am. J. Obstet. Gynecol..

[46] Leonardi M., Horne A.W., Armour M., Missmer S.A., Roman H., Rombauts L., Hummelshoj L., Wattiez A., Condous G., Johnson N.P. (2020). Endometriosis and the Coronavirus (COVID-19) Pandemic: Clinical Advice and Future Considerations. Front. Reprod. Health.

[47] Wylie J., Quinn D., Donnelly D.W., McCluggage W.G., Coleman H.G., Gavin A., McMenamin Ú.C. (2022). The Impact of the COVID-19 Pandemic on Endometrial Cancer and Endometrial Hyperplasia Diagnoses: A Population-Based Study. Am. J. Obstet. Gynecol..

[48] de Medeiros S.F., Yamamoto M.M.W., de Medeiros M.A.S., Yamamoto A.K.L.W., Barbosa B.B. (2022). Polycystic Ovary Syndrome and Risks for COVID-19 Infection: A Comprehensive Review PCOS and COVID-19 Relationship. Rev. Endocr. Metab. Disord..

[49] Wang Q., Berger N.A., Xu R. (2021). Analyses of Risk, Racial Disparity, and Outcomes Among US Patients With Cancer and COVID-19 Infection. JAMA Oncol..

[50] Cascella M., Gaudio A.D., Vittori A., Bimonte S., Prete P.D., Forte C.A., Cuomo A., Blasio E.D. (2021). COVID-Pain: Acute and Late-Onset Painful Clinical Manifestations in COVID-19-Molecular Mechanisms and Research Perspectives. J. Pain Res..

[51] Vaduganathan M., Vardeny O., Michel T., Mcmurray J.J.V., Pfeffer M.A., Solomon S.D. (2020). Renin-Angiotensin-Aldosterone System Inhibitors in Patients with COVID-19. N. Engl. J. Med..

[52] Amini M.A., Karimi J., Talebi S.S., Piri H. (2022). The Association of COVID-19 and Reactive Oxygen Species Modulator 1 (ROMO1) with Oxidative Stress. Chonnam Med. J..

[53] Nanda A., Thangapandi K., Banerjee P., Dutta M., Wangdi T., Sharma P., Chaudhury K., Jana S.K. (2020). Cytokines, Angiogenesis, and Extracellular Matrix Degradation Are Augmented by Oxidative Stress in Endometriosis. Ann. Lab. Med..

[54] Darif D., Hammi I., Kihel A., Saik I.E.I., Guessous F., Akarid K. (2021). The Pro-Inflammatory Cytokines in COVID-19 Pathogenesis: What Goes Wrong?. Microb. Pathog..

[55] Sheng J., Liu S., Wang Y., Cui R., Zhang X. (2017). The Link between Depression and Chronic Pain: Neural Mechanisms in the Brain. Neural Plast..

[56] Estes S.J., Huisingh C.E., Chiuve S.E., Petruski-Ivleva N., Missmer S.A. (2021). Depression, Anxiety, and Self-Directed Violence in Women With Endometriosis: A Retrospective Matched-Cohort Study. Am. J. Epidemiol..

[57] Moher D., Shamseer L., Clarke M., Ghersi D., Liberatî A., Petticrew M., Shekelle P., Stewart L.A., Group P.-P. (2015). Preferred Reporting Items for Systematic Review and Meta-Analysis Protocols (PRISMA-P) 2015 Statement. Syst. Rev..

[58] CRD. https://www.crd.york.ac.uk/Prospero/Display_record.Php?RecordID=356074.

[59] University of Bristol of QUADAS-2. http://www.bristol.ac.uk/Population-Health-Sciences/Projects/Quadas/Quadas-2/.

[60] Egger M., Smith G.D., Schneider M., Minder C. (1997). Bias in Meta-Analysis Detected by a Simple, Graphical Test. BMJ.

